# Amitraz poisoning: A case report of rare pesticide poisoning from Nepal

**DOI:** 10.1097/MS9.0000000000004837

**Published:** 2026-03-20

**Authors:** Nabin Pahari, Mukesh Pahari, Sagun Ghimire, Ram Bahadur Khadka, Anmol Singh Shrestha, Dipim Gautam, Samyek Dewan

**Affiliations:** a Department of Intensive Care, Lumbini Provincial Hospital, Rupandehi, Nepal; b Department of Emergency, Devdaha Medical College, Rupandehi, Nepal; c Department of Neurosurgery, B & B Hospital, Kathmandu, Nepal; d Department of Biochemistry, Crimson College of Technology, Rupandehi, Nepal; e Department of Intensive Care, Devdaha Medical College, Rupandehi, Nepal; f Department of Urology, University College London Hospital, London, UK

**Keywords:** amitraz, case report, insecticides, Nepal, poisoning

## Abstract

**Introduction and importance:**

Amitraz poisoning is a rare poisoning in agricultural and developing countries like Nepal. This case report highlights the clinical presentation and management of Amitraz poisoning.

**Case presentation:**

A 40-year-old male presented with history of vomiting and epigastric pain after suicidal ingestion of 30 mL of Amitraz (12.5% W/V). Symptoms were similar of α1 and α2 agonist including bradycardia, miosis, and hypotension. Patient required mechanical ventilation support and was given symptomatic treatment.

**Clinical discussion:**

Amitraz poisoning is rare but might be misdiagnosed for organophosphate and carbamates poisoning. All symptoms are explained by its α1 and α2 receptor agonist action.

**Conclusion:**

There is no antidote for this poisoning and treatment is solely based on symptomatic care. Normally symptoms resolve in 24–48 hours.

## Introduction

Amitraz is the most widely used pesticide and acaricide in agricultural regions such as Nepal. It is a formamidine [1, 5 di-(2, 4-dimethylphenyl)-3-methyl-1, 3-5-tri-azapenta-1, 4-diene]^[^1^]^. Amitraz poisoning in humans is uncommon and occurs through dermal, oral, or inhalational routes^[^2–5^]^. The most common route is oral, giving rise to more severe manifestations^[^6,7^]^. Few occurrences of Amitraz poisoning have been described, with symptoms primarily involving the central nervous system, including hypothermia, bradycardia, miosis, hypotension, altered sensorium, vomiting, glycosuria, hyperglycemia, and respiratory failure^[^8^]^.

Amitraz is an alpha-2-adrenergic agonist in the central nervous system (CNS), as well as an alpha-1 and alpha-2 adrenergic receptor agonist in the periphery^[^9^]^. Our case report was written in accordance with the SCARE criteria^[^10^]^. This report presents a male with a suicidal ingestion of amitraz. The symptoms developed by our patient were similar of α1 and α2 agonist including bradycardia, miosis, and hypotension. There are only a limited number of documented case reports of human intoxication and majority of them are on accidental ingestion by children^[^1–4^]^. Only a few cases of amitraz poisoning in Southeast Asia so far have been found after an extensive literature survey^[^5^]^. Therefore, there is a lack of general awareness about management of this toxin among clinicians in Asian countries^[^9^]^. This case report highlights the clinical presentation and management of Amitraz poisoning. This article is compliant with the TITAN Guidelines 2025-governing declaration and use of Artificial Intelligence (AI)^[^11^]^.

HIGHLIGHTS

- Amitraz is an alpha-2-adrenergic agonist in the central nervous system.
- But it is both alpha-1 & alpha-2 adrenergic receptor agonist in the peripheral
- The positive atropine challenge test helped rule out organophosphate poisoning i.e 1-1.2 mg of atropine causes 20–30% increase in basal heart rate.
- No antidote is present. Treatment is solely based on symptomatic management.

## Case presentation

**Figure 1.**
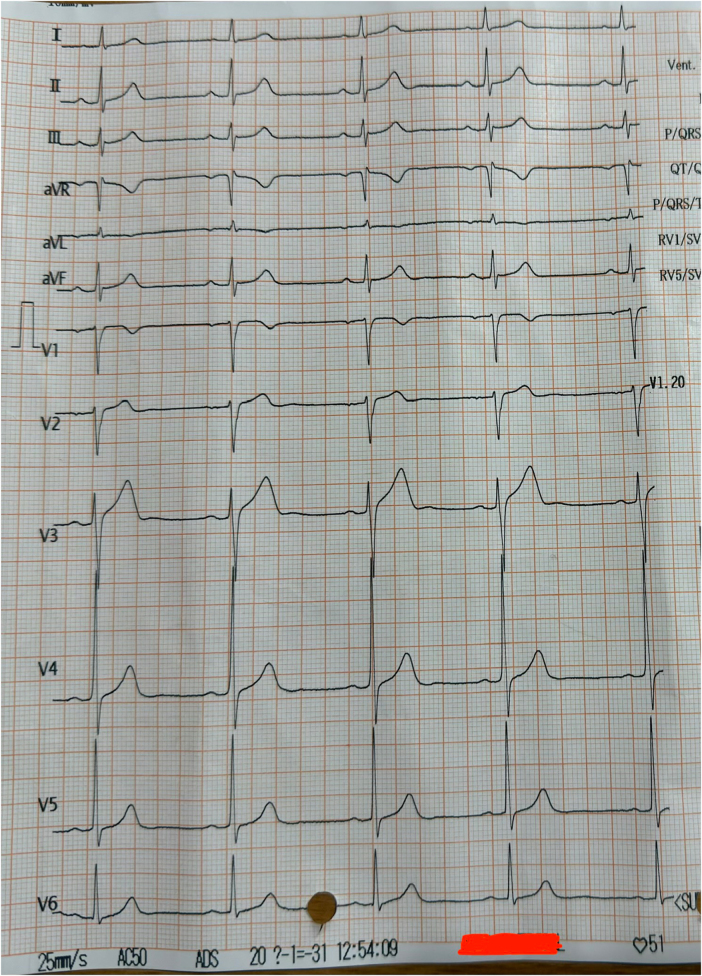
ECG of patient showing sinus bradycardia.

A 40-year-old Nepali male presented with alleged history of suicidal ingestion of 30 ml of Amitraz (12.5% W/V) following a dispute with his wife under influence of alcohol was presented to emergency room of Lumbini Provincial Hospital, Rupandehi, Nepal after 1 hour of ingestion. He had three episodes of non-projectile vomiting and pain in the epigastric region. At the time of presentation, he was fully oriented to time, place, and person. His Blood pressure was 100/70 mmHg in right brachial artery, radial pulse was 52 beats per minute, SpO_2_ was 96% in room air, respiratory rate was 15 breaths/minutes, temperature 97°F and GRBS 119 mg/dL. Twelve leads electrocardiograph (ECG) showed sinus bradycardia (Fig. 1).

**Figure 2.**
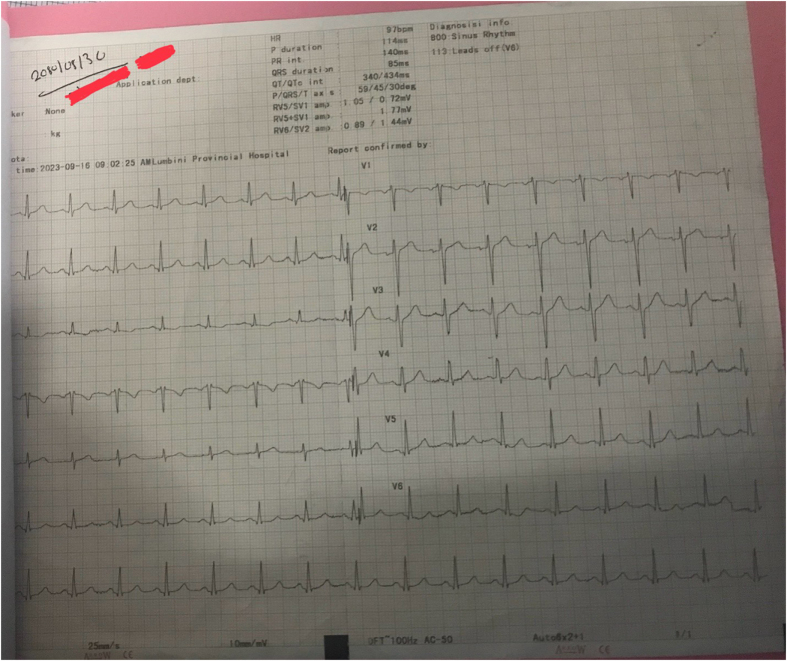
Post-atropine challenge test ECG, showing sinus tachycardia.

On examination, his both pupils were pin-point and sluggish to reaction to light. His chest had normal vesicular breath sounds on auscultation. Other systemic examination was normal. Gastric lavage was done within 30 minutes of presentation to emergency with normal saline and activated charcoal (1 g/kg), and patient was given 500 mL of 0.9% normal saline and 1.2 mg of atropine intravenously. This resulted in an increased heart rate to 114 bpm indicating atropine challenge test to be positive. An atropine challenge test is said to be negative when 1-–1.2 mg of intravenous atropine can’t increase the basal heart rate by 20–30% and in such case organophosphate poisoning is most likely in countries like Nepal. His 12 leads ECG showed sinus tachycardia (Fig. 2) post-atropine challenge test. His arterial blood gas (ABG) was normal. After 40–60 minutes of emergency presentation his Glasgow Coma Scale (GCS) dropped to E1V2M3 with respiratory rate of 9 breaths/minute. He was then intubated in the emergency room under proper sedation with intravenous ketamine and midazolam. Patient was then shifted to intensive care unit (ICU) for mechanical ventilation. As there is no known antidote for amitraz, only symptomatic treatment was done, which includes intravenous fluids, prophylactic antibiotics, sedation by intravenous midazolam and fentanyl. On the third day he was extubated as he was hemodynamically stable, and his blood parameters were normal including thyroid function test (TFT) and ABG. He had no neurological deficit and cardiovascular symptoms. On the fourth day, he was shifted to medical ward and on fifth day he was discharged from ward after psychiatric consultation. He was prescribed Sertraline and advised to follow-up in 2 weeks. After 2 weeks he came back for follow-up in the medical and psychiatric outpatient department. His basic blood investigations came normal and was treated accordingly based on depression guidelines. Patient’s lab investigations are summarized in Table 1.

**Table 1:** Lab investigations of patient pre-admission and during discharge.

Test	During admission	During discharge	Reference Range
Total Leukocyte count	12 000/mm^3^	7500/mm^3^	5000–10 000/mm^3^
Neutrophils	74%	60 %	55-70%
Lymphocytes	18%	10%	20–40%
Eosinophils	02%	0%	1–4%
Platelets	155 000	125 000	150 000–400 000/mm^3^
Amylase	40.0 IU/L	20.0 IU/L	30–220 units/L
Lipase	98.0 IU/L	30IU/L	0–160 units/L
RBS	130 mg/dL	98 mg/dL	74–106 mg/dL
Urea	20.8 mg/dL	15 mg/dL	10-20 mg/dL
Creatinine	1.9 mg/dL	1 mg/dL	0.8–1.3 mg/dL (Males: 0.6–1.2 mg/dL; females: 0.5–1.1 mg/dL)
Total bilirubin	0.5 mg/dL	0.4 mg/dL	0.3–1.0 mg/dl
Direct bilirubin	0.2 mg/dL	0.1 mg/dL	0.1–0.3 mg/dl
AST (Aspartate Aminotransferase)	96.0 IU/dL	38 IU/dL	0–35 U/L
ALT (Alanine Aminotransferase)	48.0 IU/dL	35 IU/dL	4–36 U/L

## Discussion

Amitraz acts as an alpha-2 receptor agonist in the CNS and α1 and α2 receptor agonists in the peripheral^[^12^]^. It also inhibits the monoamine oxidase (MAO) enzyme oxidase. It can also be misdiagnosed as organophospate, carbamate, since all of three have similar clinical symptoms, and hence they can be misinterpreted at times^[^9^]^. Overdose of opioids, barbiturates, benzodiazepines, phenothiazines, and tricyclic antidepressants can also display similar symptoms and signs^[^5^]^. Two human fatalities have been reported because of amitraz ingestion and one of them had ingested 6 g of the substance^[^2,13^]^. Similarly, Jorens *et al* reported a minimal hazardous dose of 3.57 mg/dL^[^5^]^. This patient ingested approximately 3750 mg orally (46.8 mg/kg).

In the majority of case reports, the onset of action occurred between 30 and 180 minutes following ingestion of substance^[^2,3^]^. Yaramis *et al* discovered that CNS depression emerged between 30 and 90 minutes and frequently resolved within 8.5 to 14 hours^[^14^]^. Furthermore, Aydin *et al* reported CNS depression in eight children that lasted 30–120 minutes and resolved in 8–18 hours^[^4^]^. But in our case, clinical symptoms started within 100–120 minutes of ingestion. Yilmaz in a case series shows that impaired consciousness was predominant with drowsiness, disorientation, and a median paediatric GCS of 9 which is similar to our case^[^2^]^. Three episodes of non-projectile vomiting, pain in the epigastric region were observed in our patient after the ingestion with both pupils as pinpoint and sluggish to reaction to light. This is consistent with several studies that have shown miosis with absence of light reflex to be the most common finding^[^2,13,15^]^.

The α1 and α2 agonistic action of amitraz poisoning causes bradycardia and hypotension^[^1^]^, which were observed in numerous case reports^[^2,3,13^]^ similar to our case. Some patients were treated with atropine for bradycardia and hypotension^[^2,4,13^]^ whereas some needed intravenous fluid for resuscitation^[^12,13^]^. Respiratory depression is also prevalent^[^4,13^]^ and in some cases, severe respiratory depression required mechanical ventilation like ours^[^2,4^]^. Similar to our case in most of the cases, no abnormality has been reported in the blood gases^[^2^]^. Respiratory alkalosis in two cases, respiratory acidosis in three cases, and metabolic acidosis in five cases were reported by Kalyoncu and colleagues^[^6^]^. This variation could be attributed to differences in monitoring techniques, ABG sampling time, and the degree of respiratory or central nervous system depression.

Hypernatremia has been rarely reported in any study^[^6,16^]^. Slight elevation in the serum ALT and AST level was observed rarely and all recovered in a few days^[^2–4^]^. In one study, the mean AST elevation was higher than mean ALT elevation^[^5^]^. In few cases, elevated alkaline phosphatase levels were seen by Ertekin and his colleagues^[^3^]^. However, the available source of evidence does not indicate any significant changes of liver and renal functions or haematological parameters associated with amitraz poisoning^[^9^]^.

No specific antidote for amitraz poisoning is present and the management is solely based on symptomatic management that includes monitoring and evaluation of cardiac, respiratory and central nervous systems^[^17^]^. Research into the role of activated charcoal has not been studied and no data comparing the effectiveness of gastric lavage and activated charcoal in relation to amitraz has been reported^[^9^]^. Yilmaz *et al* suggested performing gastric lavage only in massive doses, after endotracheal intubation to avoid inhalation or aspiration pneumonitis^[^2^]^. Patients who developed bradycardia were successfully treated with atropine^[^2^]^. Yilmaz also mentioned that using atropine is effective in cases of symptomatic bradycardia only and there is no requirement of atropine use in asymptomatic bradycardia or miosis^[^2^]^. In our study, we administered 1.2 mg of atropine intravenously, which caused the heart rate to increase to 114 bpm signifying atropine challenge test to be positive and less chance of organophosphate poisoning which is common in countries like Nepal. Intravenous fluid resuscitation and inotropic agents like dopamine or noradrenaline can be added as needed for the cases of hypotension^[^2,13^]^ and seizures respond to diazepam and lorazepam^[^2,3,13^]^. If oxygen saturation drops, oxygen should be given and some patients with severe respiratory depression need intensive care unit (ICU) stay and intubation like ours.^[^2,4,5^]^

## Conclusion

We present a 40-year-old patient with Amitraz poisoning at a dose of 46.8 mg/kg, accompanied by vomiting, bilateral miosis, hypotension, bradycardia, and respiratory depression. Amitraz’s α1 and α2 receptor agonist activity can account for all these symptoms. There is no antidote for Amitraz poisoning, thus therapy is only symptomatic. Animal studies have demonstrated that α2 adrenergic antagonists, such as Yohimbine, may alleviate symptoms; however, this represents a relatively new therapeutic concept requiring further investigation to be conducted on humans. Despite the various clinical signs and symptoms, most instances of Amitraz poisoning in humans have a positive outcome and recover within 48-72 hours of symptomatic therapy.

## Data Availability

The dissemination of the article data is freely accessed.
